# Sexual and postmating reproductive isolation between allopatric *Drosophila montana *populations suggest speciation potential

**DOI:** 10.1186/1471-2148-11-68

**Published:** 2011-03-14

**Authors:** Jackson H Jennings, Dominique Mazzi, Michael G Ritchie, Anneli Hoikkala

**Affiliations:** 1 Centre of Excellence in Evolutionary Research, Department of Biological and Environmental Science, P.O. Box 35, 40014 University of Jyväskylä, Finland; 2 ETH Zurich, Institute of Plant, Animal and Agroecosystem Sciences, Applied Entomology, Schmelzbergstrasse 9, CH-8092 Zurich, Switzerland; 3 Dyers Brae House, School of Biology, University of St. Andrews, KY16 9TH, Scotland, UK

## Abstract

**Background:**

Widely distributed species with populations adapted to different environmental conditions can provide valuable opportunities for tracing the onset of reproductive incompatibilities and their role in the speciation process. *Drosophila montana*, a *D*. *virilis *group species found in high latitude boreal forests in Nearctic and Palearctic regions around the globe, could be an excellent model system for studying the early stages of speciation, as a wealth of information concerning this species' ecology, mating system, life history, genetics and phylogeography is available. However, reproductive barriers between populations have hereto not been investigated.

**Results:**

We report both pre- and postmating barriers to reproduction between flies from European (Finnish) and North American (Canadian) populations of *Drosophila montana*. Using a series of mate-choice designs, we show that flies from these two populations mate assortatively (i.e., exhibit significant sexual isolation) while emphasizing the importance of experimental design in these kinds of studies. We also assessed potential postmating isolation by quantifying egg and progeny production in intra- and interpopulation crosses and show a significant one-way reduction in progeny production, affecting both male and female offspring equally.

**Conclusion:**

We provide evidence that allopatric *D. montana *populations exhibit reproductive isolation and we discuss the potential mechanisms involved. Our data emphasize the importance of experimental design in studies on premating isolation between recently diverged taxa and suggest that postmating barriers may be due to postcopulatory-prezygotic mechanisms. *D. montana *populations seem to be evolving multiple barriers to gene flow in allopatry and our study lays the groundwork for future investigations of the genetic and phenotypic mechanisms underlying these barriers.

## Background

The evolution of reproductive isolation between divergent conspecific populations is a key requirement for the process of speciation [1,2]. Allopatric populations in the initial stages of divergence, therefore, can provide valuable opportunities to study the onset of reproductive barriers and fertile grounds for testing hypotheses concerning the roles of environmental adaptation (divergent vs. parallel natural selection), sexual selection and random genetic divergence in generating reproductive barriers among populations. Even incomplete reproductive barriers that evolve during allopatry may play an important role in the case of secondary contact by preventing population admixture and further strengthening sexual isolation until speciation is complete [3]. Thus, identifying suitable systems (species) with divergent conspecific populations exhibiting the early signs of reproductive isolation is of great value in speciation research.

Allopatric speciation (i.e., speciation without gene flow) can occur through ecological and/or non-ecological speciation processes under natural and/or sexual selection (see e.g. [4]). Thus, relevant studies are most fruitful when carried out using species for which ample ecological, behavioral, genetic and phylogeographic data are available. Ecological speciation, also called "ecogeographic" speciation if the diverging populations are geographically isolated [5], occurs when reproductive barriers between populations evolve as a result of divergent natural selection in contrasting environments. In non-ecological speciation, genetic divergence of populations occurs through the fixation of different advantageous mutations in each population, even though they are adapting to similar environmental conditions (mutation-order model; [6]). In this case, even though the same alleles may be favored in both populations due to the similarity of their environments, they may not exhibit the same mutations or fix them in the same order, such that when the populations come back into contact, incompatible alleles may interact negatively in hybrids creating pre- and/or postzygotic reproductive barriers.

Sexual selection can contribute to reproductive isolation by, for example, driving the divergence of important male mating signals and corresponding female preferences [7-9] in particular populations and/or through sexual conflicts between the sexes (e.g. [10]). Natural and sexual selection may also work "in concert" by favoring the evolution of female sexual preferences for male ornaments that signal local adaptation, creating reproductive barriers even in the face of substantial gene flow [11]. Kirkpatrick and Ravigné [12] suggest that sexual selection is even more effective than natural selection in generating disequilibria (i.e., non-random association of alleles at two or more loci) and hence new species.

The strengths and mechanisms of various reproductive barriers and their role in speciation have been investigated in a number of recently diverged taxa, including plants [13], fungi [14], African cichlids [15], three-spined sticklebacks [16], grasshoppers [17], darters [18], walking-stick insects [19], pea aphids [20], and *Drosophila *[21]. These systems have contributed a great deal to our knowledge of speciation, however some of them are limited, for example, by a lack of knowledge of particular aspects of their natural biology, unacceptably long times since divergence (such that the speciation process is complete or nearly complete when studied) or their inability to be reared and manipulated with ease in the laboratory. Even in *Drosophila*, the number of known species with divergent populations and good background knowledge concerning their biology in nature is somewhat limited. Cactophilic *D. mojavensis *from the Sonoran Desert is one such species; mainland and peninsular Baja California populations exhibit significant premating isolation, the natural biology of the populations has been well-studied, and its full genome has been sequenced [22-24]. *D. melanogaster *has also gained attention due to evidence of significant premating isolation between Zimbabwe "Z" and cosmopolitan "M" strains [25,26] and between flies on different sides of "Evolution Canyon" [27]. However, *D. melanogaster *may not be the best choice for studies on the role of adaptation to natural environments in generating reproductive barriers, as little is known about its historical ecology in Africa, where it originated before becoming a human commensal and colonizing the world, and the causes of premating isolation remain poorly understood [28].

Recently diverged populations of the malt fly, *Drosophila montana*, with their circumpolar distribution, provide an excellent model system for tracing the onset of reproductive barriers in the early stages of speciation, as a wealth of information concerning this species' ecology, mating system, life history, genetics and phylogeography is available. The *D. virilis *group, of which *D. montana *is a member, originated in continental Asia about 20 Mya and gave rise to 12 species which now have distributions throughout the northern hemisphere, west to Fennoscandia and east to North America by way of Beringia [29]. North American and Scandinavian clades of *D*. *montana *have been isolated for between 450,000 and 900,000 years and mtDNA data suggest that there has been no recent gene exchange [30]. Adaptation to annual changes in light and temperature conditions at high latitudes and altitudes include strong photoperiodic reproductive diapause of overwintering females [31], which shows latitudinal variation (V. Tyukmaeva, personal communication), and extreme cold tolerance of both sexes [32]. Both northern and high altitude populations of this species are practically univoltine (i.e., one generation per year; [33]), while more southern populations on the west coast of North America are bivoltine [34].

Along with the abiotic factors to which populations have had to adapt, biotic factors such as interactions with other closely-related *Drosophila *species, as well as male-female coevolution and/or sexual conflict within the species may also have enhanced population divergence (see [35]). Routtu *et al*. [36] showed that Finnish, Canadian and Colorado (USA) *D. montana *populations differ in male courtship song as well as in wing and male genital morphology and that these differences do not coincide with neutral mtDNA divergence. *D. montana *females exercise strong selection on courting males based (at least) on the carrier frequency of male courtship song and female song preference has also been found to show geographic variation [37]. Furthermore, studies on sexual selection and male-female coevolution in Finnish *D. montana *suggest a conflict of interest between the sexes in the length of copulation duration [38].

The aim of the present study was to determine whether genetic and phenotypic divergence has given rise to pre- and/or postmating reproductive barriers between European (Oulanka, Finland) and North American (Vancouver, Canada) *D. montana *populations and to gain information on the potential mechanisms underlying these barriers. Using mass-bred populations from Oulanka and Vancouver, we carried out no-choice, female-choice and multiple-choice mating trials (Experiments 1, 2 and 3, respectively) to measure the strength of premating isolation between the flies of the study populations. We also investigated potential intrinsic postmating isolation by quantifying progeny production in crosses among isofemale lines and egg and progeny production in crosses involving flies from the mass-bred populations. Results of both investigations suggest significant barriers to reproduction between these focal populations.

## Results

### Mating behavior of flies in single-pair assays

**Figure 1:**
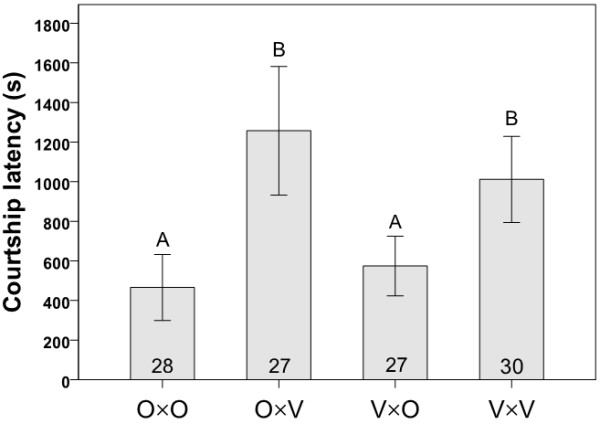
**Courtship latency**. Means ± SEs (in seconds) of the lengths of courtship latency in crosses involving flies from the same or different populations in Experiment 1. Each cross type is represented by the letters O (Oulanka) and V (Vancouver), with the female parent listed first. Sample sizes are indicated inside the bars and different letters above error bars indicate significant differences between means based on Kruskal-Wallis tests.

**Figure 2:**
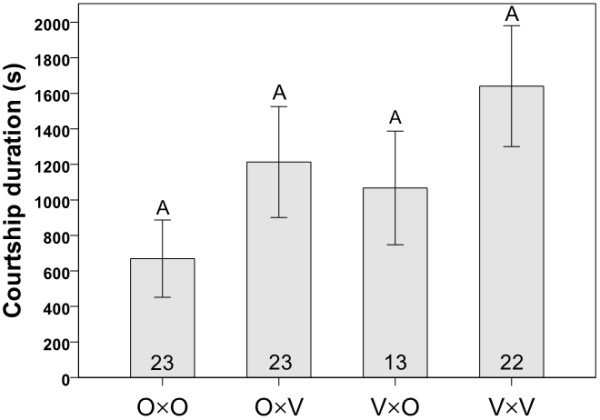
**Courtship duration**. Means ± SEs (in seconds) of courtship duration in crosses involving flies from the same or different populations in Experiment 1. Each cross type is represented by the letters O (Oulanka) and V (Vancouver), with the female parent listed first. Sample sizes are indicated inside the bars and different letters above error bars indicate significant differences between means based on Kruskal-Wallis tests.

**Figure 3:**
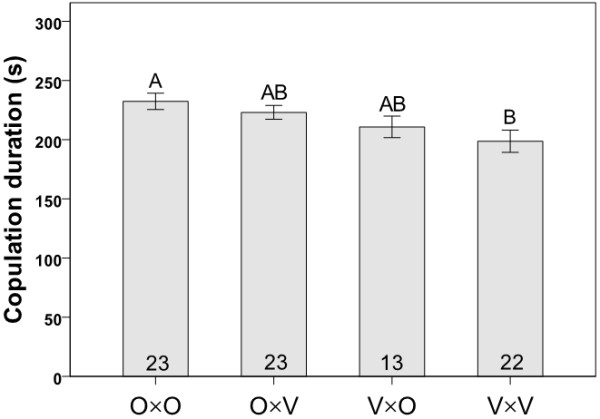
**Copulation duration**. Means ± SEs (in seconds) of copulation duration in crosses involving flies from the same or different populations in Experiment 1. Each cross type is represented by the letters O (Oulanka) and V (Vancouver), with the female parent listed first. Sample sizes are indicated inside the bars and different letters above error bars indicate significant differences between means based on Kruskal-Wallis tests.

In Experiment 1, variation in the courtship interactions of flies from mass-bred populations from Oulanka and Vancouver was traced among the four cross combinations (O×O, O×V, V×O and V×V; O = Oulanka, V = Vancouver, with females always listed first) by observing single mating pairs in individual plastic observation dishes for a maximum of two hours. Means (± SE) of the lengths of courtship latencies, courtship durations and copulation durations for the four cross types are presented in Figures 1, 2, 3, respectively. Courtship latency varied significantly among the four combinations of flies (Kruskal-Wallis: χ^2 ^= 15.650, *P *= 0.001, N = 112; Figure 1) with the longest courtships occurring in crosses involving Vancouver males (Kruskal-Wallis: χ^2 ^= 13.540, *P *< 0.001, N = 112), likely due to their overall inactivity or reluctance to court; Vancouver flies appeared generally less active in the mating chambers than flies from Oulanka. Courtship duration showed no variation among the four cross types (Kruskal-Wallis: χ^2 ^= 3.878, *P *= 0.275, N = 81; Figure 2). Copulation duration varied significantly among the four crosses (ANOVA: F_3, 81 _= 3.811, *P *= 0.013; Figure 3), with copulations being significantly longer in pure Oulanka crosses than in pure Vancouver crosses (Tukey's HSD: *P *= 0.010). This difference in copulation duration between pure population crosses may have implications for sexual conflict over the length of copulation, which has been documented in a Finnish *D. montana *population [38].

### Premating isolation

Premating isolation was measured in no-choice (single-pair), female-choice (one female, two males) and multiple-choice (30 males and 30 females from each population) situations. Sexual isolation estimates (*I_PSI_*) for the three types of mate-choice designs are presented in Table 1. *I_PSI _* values ranged between 0.132 and 0.310 and while no-choice mating trials (Experiment 1) did not yield significant estimates of sexual isolation, female- and multiple-choice trials did (Experiments 2 and 3, respectively), illustrating the importance of mate choice experimental design in testing for possible sexual isolation between closely related taxa. Also, in female-choice trials, females appeared to be more discriminatory (i.e., *I_PSI _* approached significance) when they were courted by both males instead of just one of them (*I_PSI _* = 0.249, *P *= 0.314 in trials with one courting male vs. *I_PSI _* = 0.280, *P *= 0.078 in trials where both males courted the female), which is consistent with previous work within the *D. virilis *group [39].

**Table 1:** Numbers of observed pair matings and estimates of sexual isolation across experimental designs.

		Number of matings					
Experimental Design	Number of replicate trials	OO	OV	VO	VV	*I_PSI _*± 1SD	*P *- value
Expt. 1: No-choice	122	23	23	13	22	0.132 ± 0.114	0.252
Expt. 2: Female-choice	90	23	20	9	28	0.310 ± 0.108	0.004*
Expt. 3: Multiple-choice	6	55	34	35	55	0.223 ± 0.074	0.005*

*Indicates non-random mating, i.e. significant premating isolation

### Postmating isolation

**Figure 4:**
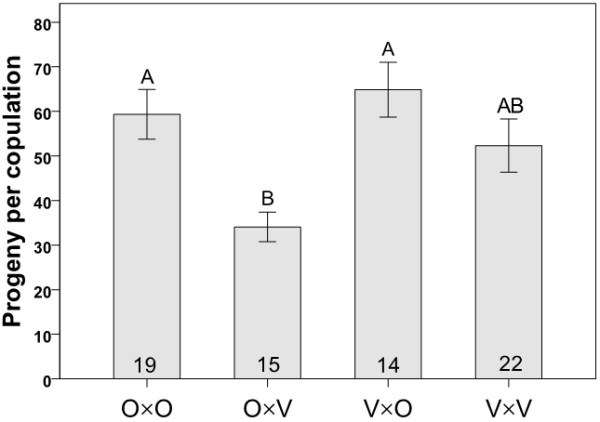
**Progeny production for mass bred populations**. Mean progeny production per female in crosses involving flies from mass-bred populations. Each cross type is represented by the letters O (Oulanka) and V (Vancouver), with the female parent listed first. Sample sizes are indicated inside the bars and different letters above error bars indicate significant differences between means based on Kruskal-Wallis tests. Error bars represent ± 1 SE.

**Figure 5:**
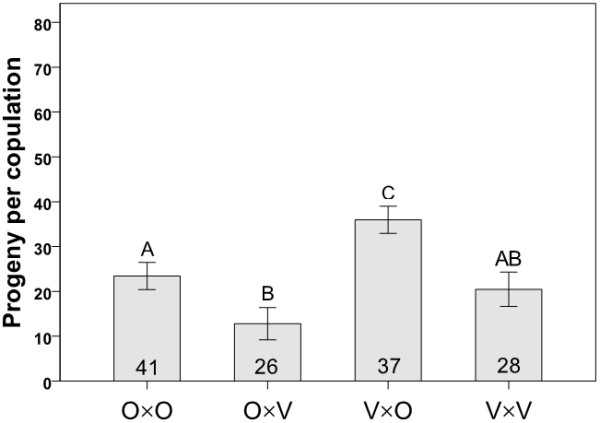
**Progeny production for isofemale lines**. Mean progeny production per female in crosses involving flies from isofemale lines. Each cross type is represented by the letters O (Oulanka) and V (Vancouver), with the female parent listed first. Sample sizes are indicated inside the bars and different letters above error bars indicate significant differences between means based on Kruskal-Wallis tests. Error bars represent ± 1 SE.

We quantified egg and progeny production for once-mated females (in all cross combinations) using a series of controlled single pair matings with flies from mass-bred populations. We also measured progeny (but not egg) production and the proportion of matings producing progeny using flies from isofemale lines. Progeny production among the four cross types showed the same general trend whether mass-bred populations or isofemale lines were used (Figures 4 and 5, respectively). In crosses with flies from mass-bred populations, progeny production varied among the four cross types (Kruskal-Wallis: χ^2 ^= 17.364, *P *= 0.001, N = 70, Figure 4) with significantly fewer progeny being produced in crosses involving Oulanka females and Vancouver males than in the reciprocal interpopulation cross (Kruskal-Wallis: χ^2 ^= 12.209, *P *< 0.0001, N = 29). Oulanka females also produced fewer progeny when mated to Vancouver males rather than their own males (Kruskal-Wallis: χ^2 ^= 11.099, *P *= 0.001, N = 34). In crosses involving isofemale lines, progeny production also showed variation among cross types (Kruskal-Wallis: χ^2 ^= 23.88, *P *< 0.001, N = 132, Figure 5) with Oulanka females again producing significantly fewer progeny when mated to a Vancouver male than when mated to a male from her own population (Kruskal-Wallis: χ^2 ^= 8.972, *P *= 0.003, N = 67). Here, interpopulation matings involving Vancouver females and Oulanka males produced more progeny per copulation than any other cross type (Kruskal-Wallis tests: *P *< 0.007 for all comparisons).

**Figure 6:**
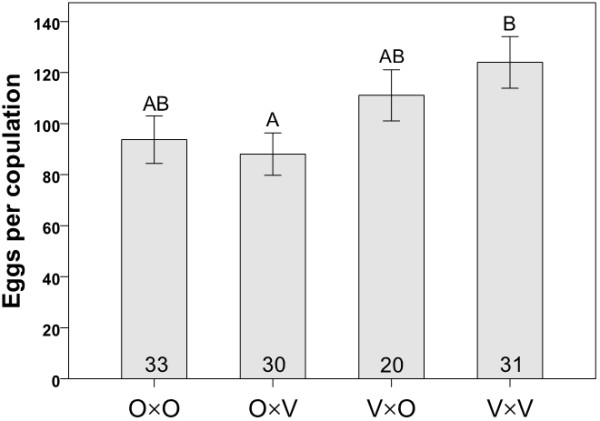
**Egg production**. Mean numbers of eggs laid by each mated female in different cross types using flies from mass-bred populations. Each cross type is represented by the letters O (Oulanka) and V (Vancouver), with the female parent listed first. Sample sizes are indicated inside the bars and different letters above error bars indicate significant differences between means based on Kruskal-Wallis tests. Error bars represent ± 1 SE.

**Figure 7:**
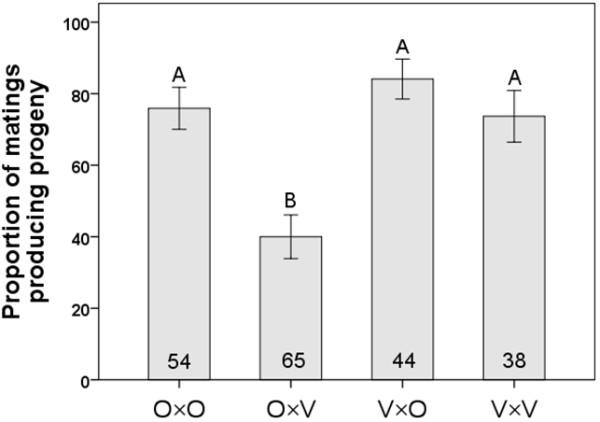
**Proportion of successful matings**. The proportion of matings that led to progeny production for the different cross types using isofemale lines. Each cross type is represented by the letters O (Oulanka) and V (Vancouver), with the female parent listed first. Sample sizes are indicated inside the bars and different letters above error bars indicate significant differences between means based on Kruskal-Wallis tests. Error bars represent ± 1 SE.

Egg production (quantified only for crosses involving flies from mass-bred populations) showed significant variation among cross types (ANOVA: F_3,114 _= 3.147, *P *= 0.028, Figure 6) with V×V crosses yielding more eggs than O×V crosses (i.e., Vancouver males were more productive with their own, as opposed to Oulanka, females; Tukey's HSD: *P *= 0.034). The same was true for the proportion of matings which produced progeny (calculated only for isofemale lines). This trait varied significantly among the cross types (Fisher's Exact Test: χ^2 ^= 29.224, df = 3, *P *< 0.0001; Figure 7) with the lowest proportion of successful copulations occurring in crosses involving Oulanka females and Vancouver males. More matings failed in this cross than in any other, which is the same combination (O×V) that produced the fewest progeny overall (Figures 4, 5, 7).

**Figure 8:**
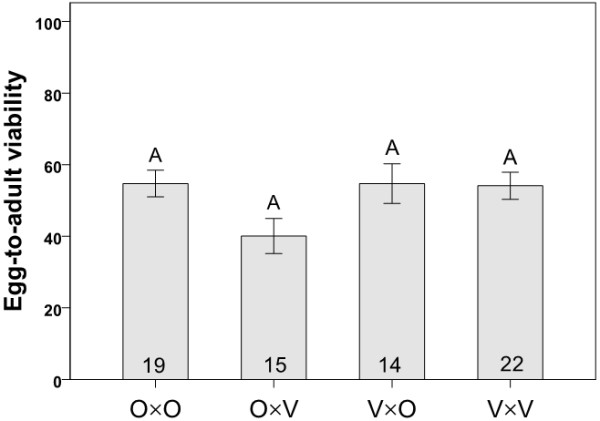
**Egg to adult viability**. Measurements of egg to adult viability of offspring resulting from crosses involving flies from mass-bred populations. Each cross type is represented by the letters O (Oulanka) and V (Vancouver), with the female parent listed first. Sample sizes are indicated inside the bars and different letters above error bars indicate significant differences between means based on Kruskal-Wallis tests. Error bars represent ± 1 SE.

There was no significant bias in offspring sex ratio in any interpopulation experimental cross (Pearson chi-square test: O×V cross, *P *= 0.559; V×O cross, *P *= 0.293 using mass bred populations; *P *= 0.599 and *P *= 0.472 for the two crosses, respectively, using isofemale lines) and thus, no evidence for Haldane's rule. And while egg-to-adult viability did not differ among cross types (Figure 8), it did show a trend similar to the proportion of copulations leading to progeny production (Figure 7) and overall progeny production (Figures 4, 5), indicating the low reproductive fitness of Oulanka females when mated to Vancouver males.

## Discussion

One of the most important goals in speciation research is to understand what kind of reproductive barriers reduce or prevent gene flow between diverging species in different speciation modes and how these barriers evolve. Sobel et al. [5] have noted that the traditional view that speciation mechanisms can be studied only in sympatric populations has led to the neglect of geographic isolation as a legitimate reproductive barrier. The authors argue that the genetically based difference in the geographic ranges of populations due to local adaptation, or "ecogeographic" isolation, is an important and often overlooked isolating mechanism. The *D. montana *populations used in this study are clearly isolated geographically. However, the genetically based differences which evolve in allopatry could be of particular importance in situations where species distribution ranges change, e.g. due to climate change, leading to secondary contact of populations.

In the present study we measured the strengths of reproductive barriers, and gained some information on the mechanisms underlying these barriers, between two geographically isolated *D. montana *populations adapted to different kinds of environmental conditions. Observations of single-pair matings showed Vancouver males to be less active than Oulanka males in courting the females, while copulation durations were longer in pure Oulanka than in pure Vancouver matings. Mating experiments revealed significant assortative mating between populations, although more work is still needed to determine on what basis females are choosing mating partners. For example, while it is known that male courtship song is important for both within-population mate choice and species recognition, and that female preferences for song traits may vary among populations [37], the role of song in between-population mate choice is unexplored. Cuticular hydrocarbon differences in this species are also poorly studied. Interpopulation crosses in this study revealed postmating reproductive barriers in the form of a one-way decrease in interpopulation progeny production.

The different experimental designs employed in this study did not always yield similar results; premating isolation was significant in experimental designs where females were able to choose between their own and foreign males, but not in no-choice situations. Choice experiments have been shown to yield higher and more realistic estimates of sexual isolation, based on evidence from the field, than no-choice experiments in other *Drosophila *species as well (e.g., [40,41]). Our data are also concordant with previous work with *D. montana *which demonstrated that female discrimination is stronger when the females are provided with a choice of mates [39]. In nature, flies of this species may occasionally encounter problems finding mates when population densities are low, so females may exercise choice when they have a possibility to do so and accept less-favoured males when there are no "better" ones available [39].

While allopatric speciation can occur rapidly by divergent natural and sexual selection between conspecific populations adapted to live in different environments, it may proceed more slowly when the populations occur in more uniform surroundings (under uniform natural selection [[42], but see [43]]). *D. montana *occupies boreal riparian habitats throughout its distribution, carrying out its life cycle near water in the moist, decaying plant tissues of various alder, birch and aspen tree species [29]. While their habitats are in many ways similar on either side of the Atlantic, abiotic factors like daily and seasonal light and temperature regimes show strong differences between Vancouver and Oulanka. According to Schemske [44], these kinds of environmental factors are stronger sources of selection than biotic interactions in temperate species. Oulanka is located at a much higher latitude (66°N) than Vancouver (49°N) and in nature Oulanka females spend the cold and long winters in photoperiodic reproductive diapause, producing only one generation per year [31]. Vancouver females also spend the winter in diapause, but enter into diapause much later in autumn and emerge earlier in the spring; thus, they can have at two or more generations per year [34]. The effects of this difference in the speed of evolution on reproductive isolation have not been explored.

There are also differences in species assemblages between the two populations. In Vancouver, *D. montana *is the most abundant *D. virilis *group species, with one other species (*D. flavomontana) *being found only occasionally (M. Ritchie, personal communication, also see [45]). Oulanka, on the other hand, is currently home to three sympatric (and cryptic) *D. virilis *group species - *D. ezoana, D. littoralis*, and *D. Montana *(also, historically, *D. lummei*) - that utilize the same resources and are often found at the same riparian lekking sites [29]. Interspecific courtships are quite frequent in the wild although the species do not hybridize [46]. This results in qualitative differences in the arena of sexual selection in each population. Thus, the evolution of *D. montana *in areas of sympatry with other closely related species (as in Oulanka) may have placed different selective pressures on male mating signals, female choosiness, or both.

Sexual selection has long been regarded as a possible engine driving divergence among isolated populations by shifting male mating signals and corresponding female preferences [47,48] and sexual conflict, which occurs when the genetic interests of males and females diverge, has also gained recent attention in this context [10]. If sexual conflict arises (or evolves) independently in geographically isolated populations, it is easy to imagine that divergent phenotypic and genetic change might follow. In a recent study, Mazzi et al. [38] showed that in Finnish *D. montana *sexual conflict arises in the length of copulation duration, which is primarily under female control. Towards the end of copulation, the female vigorously attempts to dislodge the male by kicking him with her hind legs, while the male struggles to extend the copulation. The authors concluded that the main cost to females and benefit to males of a prolonged copulation is the extended latency to female remating, consistent with the 'extended mate guarding hypothesis' [49,50]. Our finding that copulation duration is significantly longer in crosses involving Oulanka females and males than in crosses involving Vancouver females and males suggests that the degree of sexual conflict over this trait may differ between the two populations.

Interpopulation matings involving Oulanka females and Vancouver males produced significantly fewer progeny than other crosses, although in this study we did not determine at which stage (e.g, sperm transfer/storage, cryptic female choice, sperm-egg interaction, embryonic/larval/pupal development) the observed hybrid dysfunction occurred. We did find, however, that both sexes of offspring resulting from O×V crosses were affected, not just one. Haldane's rule states that in crosses between divergent taxa, when one sex of the offspring is either sterile or inviable, it is the heterogametic sex. It is generally thought that in early stages of divergence, intrinsic postzygotic isolation due to classic Dobzhansky-Muller incompatibilities almost always affects only the heterogametic sex first, obeying Haldane's rule, and that hybrid problems affecting both sexes usually appear only later on in the speciation process [43]. Coyne and Orr [51] have argued that Haldane's rule is "nearly ubiquitous" in the early stages of speciation, basing their claim on the fact that they have observed no cases where both sexes were sterile or inviable in only one direction of hybridization between taxa with low genetic distances, as we have shown here. If the postmating isolation between Oulanka females and Vancouver males is indeed post *zygotic*, it could stand as one exception to their observation.

Perhaps a more plausible explanation is that the low offspring production in these crosses represents a postcopulatory-prezygotic (PCPZ) mechanism, rather than a postzygotic one. Markow [52] has shown that *Drosophila *spp. which remate rapidly exhibit higher levels of PCPZ sexual selection than those that do not. Rapid remating results in more sperm overlap in the female reproductive tract, allowing selection to favor ejaculate traits which increase fertilization success or cryptic female choice. Since Finnish *D. montana *females remate rapidly and are known to exhibit high levels of multiple insemination in nature [53], it may be that in this population particular ejaculate traits, such as male accessory gland proteins (ACPs), have been under strong selection. The potential role of male ACPs and/or PCPZ isolation, e.g. 'contypic' sperm precedence (see [54]), in *D. montana *deserves further investigation, particularly in light of the surprisingly high number of progeny produced from the reciprocal (V×O) cross. Furthermore, as Matute and Coyne [55] have shown, reduction in hybrid viability may manifest itself in other, previously unacknowledged forms, such as reduced female longevity after heterotypic matings and increased hatching intervals and egg-to-adult development time in hybrid offspring, illustrating the need for more work in this area.

## Conclusion

Identifying which isolating mechanisms are first to evolve during population divergence and which are most important in allowing recently diverged taxa to remain genetically distinct remain important questions in the field of speciation research. The finding that divergence between *D. montana *populations has occurred to the extent that significant pre- and postmating reproductive barriers have evolved provides a valuable opportunity to trace the onset of these barriers in the earliest stages of species diversification. Indeed, Finnish *D. montana *was originally described by Lakovaara and Hackman [56] as a separate species, *D. ovivororum*, and North American *D. montana *populations have been traditionally divided into three forms - standard, Alaskan-Canadian, and giant - based on inversion frequencies, body size and geographic location [28].

Our data lay the groundwork for studies which should aim to not only measure the strengths of potential reproductive barriers, but also identify the mechanisms, both genetic and phenotypic, underlying these barriers. Whether these populations are diverging by ecological or mutation-order processes, and whether the reproductive barriers reported here would be sufficient to prevent fusion in sympatry, remain to be explored. Future work on *D. montana *will incorporate more study populations and test hypotheses concerning the role of various potential PCPZ and postzygotic mechanisms of isolation. In general, speciation studies should focus on disentangling the causal connections between natural selection, sexual selection, drift and reproductive isolation and trace the corresponding genetic changes that are ultimately responsible for maintaining isolating barriers between divergent taxa.

## Methods

### Flies and rearing procedures

Mating experiments were carried out in Spring and Summer 2009 using genetically variable mass-bred populations established by combining the F3 progenies (20 males and 20 females) of 20 isofemale lines whose founders were collected in Summer 2008 in Oulanka, Finland (≈ 66°N) and Vancouver, Canada (≈ 49°N). Once established, the laboratory populations were maintained for two to four generations with several hundred flies per cage (25×25×60 cm wooden box with a clear plexiglas top and 8 available food bottles) before offspring were collected for use in mating experiments. We also used four isofemale lines per population (see *Egg and progeny production *below) which were established from the progenies of single females collected in the wild from the same locations as described above in Oulanka and Vancouver in Summer 2003. Here, single-pair matings were carried out in three time blocks within two years of the establishment of the lines in the laboratory (June 2004, August 2004, and July 2005).

All flies were maintained in continuous light at 19 ± 1°C and 65 ± 5% relative humidity to ensure that females reached sexual maturity instead of entering reproductive diapause. All experimental flies were reared from the eggs laid by females on malt medium in half-pint bottles. Flies were separated by sex within three days of eclosion to ensure virginity and were kept in sex-specific groups of 5 to 10 in fresh food vials until sexually mature, 21-28 days post-eclosion (except for multiple-choice experiments, where flies were kept in groups of 30-35 individuals). Flies were used only once in any mating experiment.

European and North American *D. montana *populations are morphologically indistinguishable so they were marked for identification where necessary. For marking, flies were placed in fresh vials on food coloured with one drop of either blue or red food colouring, 12-24 hours prior to being used in mating experiments (as in [25,41]). The colours were alternated between populations as the trials proceeded to control for potential marking effects. The origin of each fly was identified by dissection based on the colour of its intestinal contents.

### Mating experiments

All mating experiments were conducted between 8:00 and 11:00 a.m. at 20 ± 1°C at the University of Jyväskylä. In no-choice trials, various aspects of mating behaviour were observed and recorded, and for all experiments, an index of sexual isolation was calculated based on the relative numbers of matings between flies in different pair combinations using the program JMating [57,58].

#### Experiment 1: No-choice tests

For each trial, one female and one male were transferred into a gauze-covered plastic dish (diameter 5 cm, height 0.7 cm) with a piece of moistened filter paper covering the floor. The behaviour of the flies was observed until the end of copulation (if the mating was successful) or until two hours had elapsed. For each individual pair of flies, we recorded the lengths of courtship latency, courtship duration and copulation duration. Courtship latency was measured from the time when the flies were transferred into the chamber until the male began to court the female, indicated by male following, tapping, wing vibration and/or licking. Courtship duration was measured from the beginning of courtship to the onset of copulation, when the male mounted the female and locked genitalia. Copulation duration was measured from the beginning of copulation to the end of it, when the male fully disengaged from the female. At least 30 replicates were carried out for each of the four possible pair combinations (O×O, O×V, V×O and V×V) resulting in 122 total trials.

#### Experiment 2: Female-choice tests

These trials were performed in the same way as Experiments 1, except that for each trial a single female was transferred to the plastic dish together with two males - one from each population. While this type of experimental design does allow for the assessment of mate choice (i.e., strength of sexual isolation), it is not amenable to measuring individual fly behaviour in detail as in Experiment 1. Males were marked for identification in Experiment 2, which involved altogether 90 female-choice trials (45 per female type).

#### Experiment 3: Multiple-choice tests

For each replicate mating trial in Experiment 3, 120 flies (30 males and 30 females from each mass-bred population) were introduced into a clear 6×6×6 cm Plexiglas chamber and allowed to court and mate until half of the possible matings (30 of 60 pairs) occurred or until one hour had elapsed (although no trials required the full one hour to produce 30 mated pairs; see [59] for a statistical justification of this method). Both females and males were marked for identification and copulating pairs were removed with an aspirator and subsequently dissected. On three occasions, a fly could not be identified due to a lack of color in the intestine, so these mating pairs were removed from the analysis. The mating chamber was washed thoroughly between trials to remove any potential pheromones remaining from flies in the previous experiment. This experiment involved six replicate trials, resulting in the identification of 177 copulating pairs.

### Egg and progeny production

#### Mass-bred populations

We carried out a separate set of single pair matings using flies from the mass-bred populations and quantified both eggs and emerging offspring (males and females) among the four cross types. As in Experiment 1, individual pairs of virgin, sexually mature flies were combined allowed to copulate in gauze-covered petri-dishes. After mating, females were transferred singly into malt vials and allowed to lay eggs. After three days, they were transferred again into fresh malt vials and discarded after a total of seven days of oviposition, since once-mated *D. montana *females are known to produce progeny for approximately six days [53]. Eggs laid on the surface of the food were counted under a dissecting microscope and progeny were counted every other day as they emerged until eclosion ceased. At least 14 replicates were made for each cross type, resulting in total of 70 pair matings.

#### Isofemale lines

To study progeny production in isofemale lines and their crosses, we made single-pair crosses between females and males of the four isofemale lines per population in all possible combinations (a 64 cross, diallel design) as described above. Numbers of female and male progeny produced by each mated female were counted and coded as an intrapopulation (O×O or V×V, whether within or between strains) or interpopulation (O×V or O×V) cross for statistical analysis. Only fertile copulations (132 total replicates) were included in this dataset and eggs were not counted. We also obtained from this experiment the proportion of copulations that were successful, i.e., lead to progeny production, for each cross type.

### Statistical analysis

#### Behavioural analysis

In Experiment 1, we used Kruskal-Wallis tests to analyze courtship latency and courtship duration, which were non-normally distributed. In these non-parametric analyses, we first looked for variation among the four cross types, and then, if the variation was significant, we made pairwise comparisons among the different cross types, controlling for multiple tests. Copulation duration showed a normal distribution and was analyzed using one-way ANOVA and post-hoc Tukey's HSD with "cross type" (four levels) as the main factor. In cases where multiple tests were made using single datasets, alpha levels were adjusted using step-down sequential Bonferroni correction. We report results as "significant" only if they remain so after adjustment. All statistical analyses were performed with SPSS v 16.0 (SPSS Inc., Chicago, Illinois, USA).

#### Premating isolation

For each mate choice design we used the program JMating to calculate the index of sexual isolation *I_PSI _* [57,58]. *I_PSI _* ranges from -1 to 1, where 0 represents random mating, +1 represents complete assortative mating (i.e., all matings are homotypic) and -1 represents complete disassortative mating (i.e., all matings are heterotypic). Statistical significance of sexual isolation was determined by bootstrapping 10,000 times in JMating.

#### Postmating isolation

The data for egg production in crosses involving flies from the mass-bred populations were normally distributed, so a one-way ANOVA and post-hoc Tukey's HSD were used to identify differences among cross types. Progeny production in the same experiment was non-normally distributed so Kruskal-Wallis tests were used. We also calculated egg-to-adult viability for each female that produced progeny by simply dividing the number of adult progeny produced by the number of eggs laid. It should be noted, however, that this only gives a rough estimate for egg-to-adult viability, as females do lay unfertilized eggs and it was not possible to distinguish these from the fertilized ones as the eggs were being counted.

Progeny production resulting from matings involving females from the isofemale lines showed no significant variation over the three time blocks (Kruskal-Wallis: χ^2 ^= 2.207, df = 2, *P *= 0.332) so the data were pooled for analysis. Statistical significance of differences among cross types was determined with Kruskal-Wallis tests, since the data were again non-normally distributed. Alpha levels were adjusted for multiple testing with step-down sequential Bonferroni correction where necessary. Differences in the proportion of matings producing progeny among the different cross types were analyzed using Fisher's Exact Test.

## Authors' contributions

JJ designed and carried out all experiments using mass-bred populations, analyzed the data and wrote the bulk of the manuscript. DM and AH designed and conducted the experiments with isofemale lines and analyzed the data. MR helped with data analysis and provided valuable ideas concerning the study in general throughout its various stages. AH also guided the project and contributed a great deal to the final text. All authors read and approved the final manuscript.
